# The Expression of Amino Acid and Fatty Acid Receptors Show an Age-Dependent Pattern Involving Oral Cavity, Jejunum and Lower Gut Sensing in Broiler Chickens

**DOI:** 10.3390/ani13193120

**Published:** 2023-10-06

**Authors:** Paloma Cordero, Francisca Díaz-Avilés, Paulina Torres, Miguel Guzmán, Shahram Niknafs, Eugeni Roura, Sergio A. Guzmán-Pino

**Affiliations:** 1 Programa de Doctorado en Ciencias Silvoagropecuarias y Veterinarias, Campus Sur, Universidad de Chile, Santiago 8820808, Chile; paloma.cordero@veterinaria.uchile.cl; 2 Departamento de Fomento de la Producción Animal, Facultad de Ciencias Veterinarias y Pecuarias, Universidad de Chile, Santiago 8820808, Chile; francisca.diaz@ug.uchile.cl; 3 Laboratorio de Patología Aviar, Facultad de Ciencias Veterinaria y Pecuarias, Universidad de Chile, Santiago 8820808, Chile; paulinatorrescelp@gmail.com (P.T.); mguzmanm@udla.cl (M.G.); 4 Nucleus of Applied Research in Veterinary and Agronomic Sciences, Faculty of Veterinary Medicine and Agronomy, Universidad de las Américas, Santiago 7500975, Chile; 5 Centre for Nutrition and Food Sciences, Queensland Alliance for Agriculture and Food Innovation, The University of Queensland, St Lucia, QLD 4072, Australia; s.niknafs@uq.edu.au (S.N.); e.roura@uq.edu.au (E.R.)

**Keywords:** amino acids, broiler chicken, fatty acids, gastrointestinal tract, gene expression, nutrient sensors

## Abstract

**Simple Summary:**

There is limited information regarding dietary nutrient sensors in chickens. This study explored the expression of amino acid (AA) and fatty acid (FA) sensors in the gastrointestinal tract (GIT) of broilers. This research focused on quantifying gene expression of AA and FA sensors in ten tissues, including the upper, middle, and lower GIT sections of 7 and 26-day-old birds. Early in life (7 days), chicks develop a high sensing capability in the oral cavity for AA and the cecum and colon for short-chain fatty acids, presumably related to bacterial fermentation. In turn, at 26 days of age, a strong emergence of the role of the jejunum sensing AA and FA occurs, indicating the major role of the middle GIT section in nutrient digestion, absorption, and the gut-brain axis.

**Abstract:**

This work aimed to evaluate the gene expression of amino acids (AA) and fatty acids (FA) sensors in the gastrointestinal tract (GIT) of chickens at two different ages (7 and 26 days post-hatch). Sixteen broilers (Ross 308) were selected, and ten sections of the GIT, including upper (tongue base, upper palate, crop, proventriculus), middle (gizzard, duodenum, jejunum, ileum), and lower GIT section (cecum, colon) were collected for analysis. Relative gene expression of AA (T1R1, T1R3, mGluR1, mGluR4, CaSR, GPR139, GPRC6A, GPR92) and FA (FFAR2, FFAR3, FFAR4) sensors were assessed using qPCR. The statistical model included age, GIT section, and gene. In addition, the correlations between gene expressions were calculated. At day 7, a significantly (*p* = 0.004) higher expression of AA sensors in the oral cavity and FA sensors in the lower GIT section (i.e., cecum and colon) compared to the middle section was recorded. A higher expression of AA compared to FA sensors was detected at the upper GIT section in 7 (*p* < 0.001) and 26-day-old chickens (*p* = 0.026). Thus, at day 7, AA sensors were predominantly (*p* < 0.05) expressed in the upper GIT section (mainly oral cavity), while FA sensors were mainly expressed in the lower GIT section, at cecum (FFR2 and 4) or colon (FFAR3). These results may indicate that in early life, both ends of the GIT are fundamental for feed intake (oral cavity) and development of the microbiota (cecum and colon). In contrast, at 26 days of age, the results showed the emergence of both AA and FA sensors in the jejunum, presumably indicating the essential role of the jejunum in the digestion absorption of nutrients and the signaling to the brain (gut-brain axis) through the enteroendocrine system. Significant positive correlations were observed between T1R1 and T1R3 (r = 0.85, *p* < 0.001), CaSR and T1R1 (r = 0.78, *p* < 0.001), CaSR and T1R3 (r = 0.45, *p* < 0.050), and mGluR1 and FFAR3 (r = 0.46, *p* < 0.050). It is concluded that the gene expression is greater in the oral cavity for AA sensors and the lower gut for FA sensors. On day 26, the role of jejunum regarding nutrient sensing is highlighted.

## 1. Introduction

The primary function of the gastrointestinal tract (GIT) is the digestion of foods and the absorption of nutrients. Nutrient sensing plays a crucial role in communicating with the brain, mainly through releasing gut peptides, which ultimately control dietary selection and the hunger/satiety cycle [1]. Thus, the ability to monitor the chemical nature of luminal contents is essential to adapt motility, digestive enzyme and gut peptide secretions, and expression and mobilization of nutrient transporters [2,3]. The G-protein coupled receptors (GPCRs) associated with the taste system in the oral cavity have also been found in the GIT, responding specifically to dietary nutrients such as amino acids (AA) and fatty acids (FA) [4,5]. While most of the GIT chemosensory principles have been described in mammals, in recent years, a plethora of information has been uncovered on the avian taste system [6,7,8]. Previous studies have suggested that nutrient sensors are differentially expressed when comparing different stages of embryonic and post-hatching development, indicating changes in nutrient sensing mechanisms and, ultimately, digestive function [7,8,9,10,11,12].

The perception of dietary peptides, AA, and nucleotides in avian species is mediated by a group of GPCRs, mainly the heterodimer transmembrane receptor consisting of two proteins of the family 1 of taste receptors, the T1R1 and T1R3 (taste 1 receptor member 1 and 3), the metabotropic glutamate receptors 1 and 4 (mGluR1 and mGluR4), and the calcium-sensing receptor (CaSR) [11,13]. In addition, other GPCRs associated with peptide and AA sensing have been reported in mammals, such as the G protein-coupled receptor 92 (GPR92), the G protein-coupled receptor family C group 6 member A (GPRC6A), and the G protein-coupled receptor 139 (GPR139), where the functional relevance in avian species is lacking to date [7,14]. In addition, chickens are highly responsive to dietary fats [15]. The perception of dietary fats is mediated by free FA receptors (FFAR), including FFAR2, FFAR3, and FFAR4 [7,10]. However, there is no evidence of a systematic study assessing the gene expression of AA and FA sensors along the complete GIT of broilers because studies on nutrient sensors in chickens have focused on gene expression analysis of a limited number of receptors and tissues, mainly the oral cavity. This study aimed to evaluate the expression of previously reported avian sensory receptors in ten different sections of the chicken GIT and to determine the impact of two different stages of broiler development on the expression of the genes of interest. In addition, potential correlations between their expressions were also analyzed.

## 2. Materials and Methods

### 2.1. Animals, Housing, and Tissue Sampling

Animals used in this study were obtained from a field trial that included 96 one-day-old male broiler chickens (Ross 308). Birds were distributed in groups of three chickens per pen in the Experimental Unit for Poultry Nutrition and Production of the Faculty of Veterinary and Animal Sciences (FAVET) of the University of Chile (UCH). This research facility has a conventional structure with natural ventilation and 32-floor pens with wood-shaving beds. It is heated by gas brooders with temperature control and has automated drinkers and individual feeders arranged in each pen. Environmental conditions of temperature, relative humidity, and lighting programs were set up according to the genetic strain guidelines. Feed and water were offered ad libitum. The feeding program consisted of starter and grower commercial diets formulated to meet or exceed all nutrient requirements set by the NRC (1994), and the guidelines set by the breeder [16] were offered to chickens from 1 to 23 and 24 to 42 days, respectively (Table 1).

On days 7 and 26 post-hatch, one healthy chicken from each pen (*n* = 8) was selected based on body weight as close as possible to the average of the pen for tissue sampling. The remaining two chickens in each pen were used to develop feeding behavior tests not reported in this study. Chickens were sacrificed using manual cervical dislocation [17] at the necropsy room of the Avian Pathology Laboratory of FAVET, to be later processed at the Centralized Veterinary Research Laboratory of FAVET. Tissue samples were collected from ten different sections along the GIT, including the upper (tongue base, upper palate, crop, proventriculus), middle (gizzard, duodenum, jejunum, ileum), and lower tract (cecum, colon). Samples were processed to determine the relative expression of AA and FA sensing genes by quantitative PCR (qPCR). The AA sensor genes studied were T1R1, T1R3, mGluR1, mGluR4 (linked to L-glutamic acid perception), CaSR, GPR139 (L-phenylalanine and L-tryptophan), GPRC6A (other L-amino acids), and GPR92 (peptides). The FA sensor genes studied were FFAR2, FFAR3 (short-chain fatty acids), and FFAR4 (medium and long-chain fatty acids).

### 2.2. mRNA Isolation, cDNA Synthesis, and qPCR

Tissue samples were homogenized with D1000 Homogenizer (Benchmark Scientific, Sayreville, NJ, USA) and fixed with β-Mercaptoethanol and a lysis buffer (Thermo Fisher Scientific, Pleasanton, CA, USA). Total RNA was extracted using a GeneJET RNA Purification Kit (Thermo Fisher Scientific, Pleasanton, CA, USA) according to the manufacturer’s instructions. Total RNA was quantified using a Qubit 3.0 (Fluorometer, San Diego, CA, USA). Genomic DNA was removed using a DNase I Kit (Thermo Fisher Scientific, Pleasanton, CA, USA), and the cDNA was synthesized using an Affinity Script Q-PCR cDNA Synthesis Kit (Agilent Technologies, Santa Clara, CA, USA) in a TC1000-G gradient thermal cycler (SciLogex, Rocky Hill, CT, USA). Gene-specific primers were used according to the published cDNA sequences to assess the relative mRNA expression of AA and FA sensors and the housekeeping gene β-actin in the different tissues. Primers were designed with the aid of Primer3Plus (version 2.3.1, Free Software Foundation, Boston, MA, USA) and are listed in Table 2. Amplification efficiency between all primers and probe sets was confirmed using serial 3-fold dilutions of cDNA from GIT tissues. The ten μL qPCR reaction consisted of 2X Brilliant II SYBR Green QPCR master mix (5 μL; Agilent Technologies, Santa Clara, CA, USA), target forward primer (200 nM), target reverse primer (200 nM), synthesis reaction cDNA (1 μL), and PCR grade nuclease-free water to adjust the final volume. The qPCR amplification was carried out in an Eco Real-Time q-PCR System (Illumina Incorporated, San Diego, CA, USA), and the thermal cycling conditions were 95 °C for 10 min, followed by 40 repetitive cycles at 95 °C for 30 s and 60 °C for 1 min. All reactions were performed in triplicates. For each reaction, the level of gene expression was recorded as cycle threshold (CT) values corresponding to the number of cycles where the fluorescence signal can be detected above a threshold value. The CT averages for each biological replicate were calculated and transformed into relative values through the ∆∆CT formula [18].

### 2.3. Statistical Analysis

The Kruskal–Wallis test was used to analyze the data considering the fixed effects of chickens’ age, GIT section (i.e., upper, middle, and lower), tissue, sensor, age × sensor, tract × sensor, and age × GIT section × sensor. Dunn’s multiple comparison test was used to determine the difference between medians. between gene expressions were also analyzed with the Spearman correlation coefficient test. Values with a *p* ≤ 0.050 were considered significantly different. All analyses and figures were conducted with RStudio software (version 4.1.3, Boston, MA, USA) and GraphPad Prism (version 8.0.2, Boston, MA, USA).

## 3. Results

### 3.1. Differential Expression of AA and FA Sensors between 7 and 26 Days Post-Hatch

**Figure 1:**
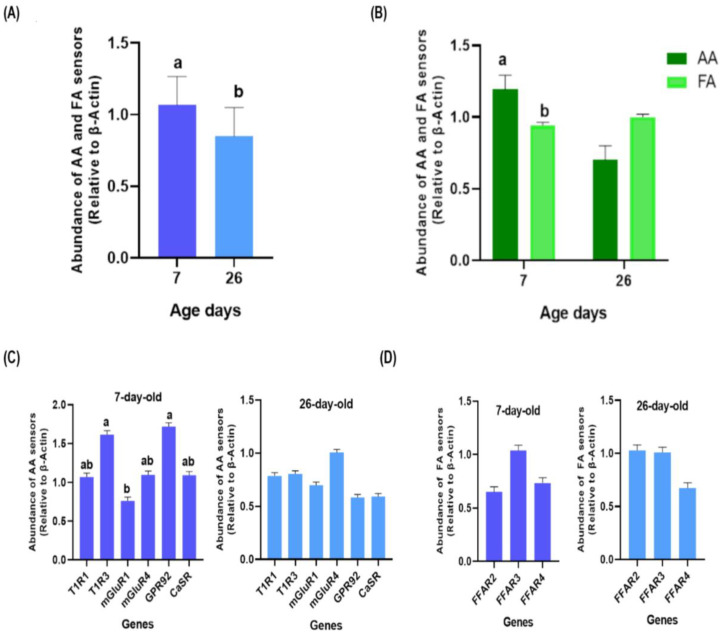
Gene expression of amino acids (AA) and fatty acids (FA) sensors in all tissues sampled according to the age of broiler chickens. Gene expression of AA and FA sensors in (**A**) 7 and 26-day-old birds, and (**B**) according to sensors group. (**C**) Gene expression of AA (T1R1, T1R3, mGluR1, mGluR4, GPR92, and CaSR) sensors in 7 and 26-day-old chickens. (**D**) Gene expression of FA (FFAR2, FFAR3, and FFAR4) sensors in 7 and 26-day-old chickens. Dark green bars indicate gene expression of AA sensos and light green bars of FA sensors. Dark blue bars indicate gene expression on 7-day-old chickens and light blue bars on 26-day-old chickens. (a, b) = significant differences (*p* ≤ 0.050).

A higher expression of the AA and FA sensors groups was observed in the 7 compared to 26-day-old chickens (*p* < 0.050; Figure 1A). The AA were significantly (*p* < 0.001) more expressed than the FA sensors at 7 days, while no significant differences (*p* = 0.066) were detected at day 26 (Figure 1B). When comparing the gene expression of AA sensors, a higher expression of T1R3 and GPR92 than mGluR1 was determined in 7-day-old chickens (*p* = 0.014), while no differences (*p* = 0.417) were observed in 26-day-old broilers (Figure 1C). There were no differences in the relative abundance of the three FA receptors FFAR2, 3, and 4 at day 7 (*p* = 0.628) or day 26 (*p* = 0.158; Figure 1D).

### 3.2. Differential Expression of AA and FA Sensors between Upper, Middle, and Lower GIT

**Figure 2:**
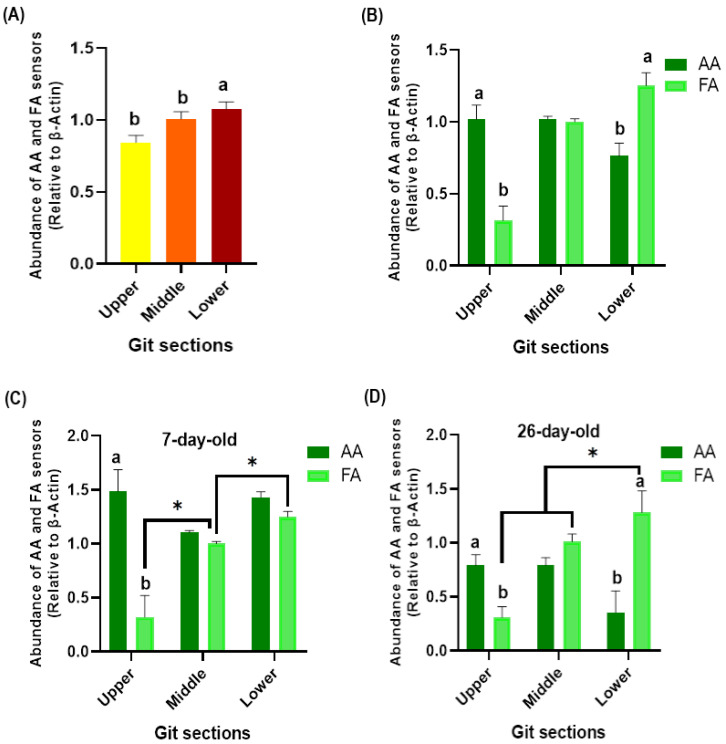
Gene expression of amino acid (AA) and fatty acid (FA) sensors according to the GIT section of broiler chickens, (**A**) at upper, middle, and lower sections of birds, (**B**) and according to sensors group. Gene expression of AA and FA sensors at upper, middle, and lower GIT sections (**C**) in 7 (**D**) and 26-day-old chickens. Yellow, orange and red bars indicate gene expression on GIT section. Dark green bars indicate gene expression of AA sensos and light green bars of FA sensors. (a, b) = significant differences within GIT sections (*p* ≤ 0.050). (*) = significant differences between GIT sections (*p* ≤ 0.050).

Overall, a higher expression of AA and FA sensors was found in the lower GIT compared to the middle and upper sections of birds (*p* = 0.004; Figure 2A). No differences between the AA and FA sensors were detected in the middle GIT section of birds (*p* = 0.368; Figure 2B). A greater expression of AA than FA sensors was detected at the upper GIT in 7 (*p* < 0.001; Figure 2C) and 26-day-old chickens (*p* = 0.026; Figure 2D). On the contrary, a greater expression of FA than AA sensors was found in the lower GIT of 26-day-old chickens (*p* < 0.001; Figure 2D). When analyzing the differential expression of the AA and FA sensors between GIT sections, no differences were observed concerning AA sensors (*p* > 0.050). However, a higher expression of FA sensors was found at lower GIT compared to the middle and upper sections in 7 (*p* < 0.001) and 26-day-old chickens (*p* < 0.001).

### 3.3. Differential Expression of AA and FA Sensors between GIT Tissues

**Figure 3:**
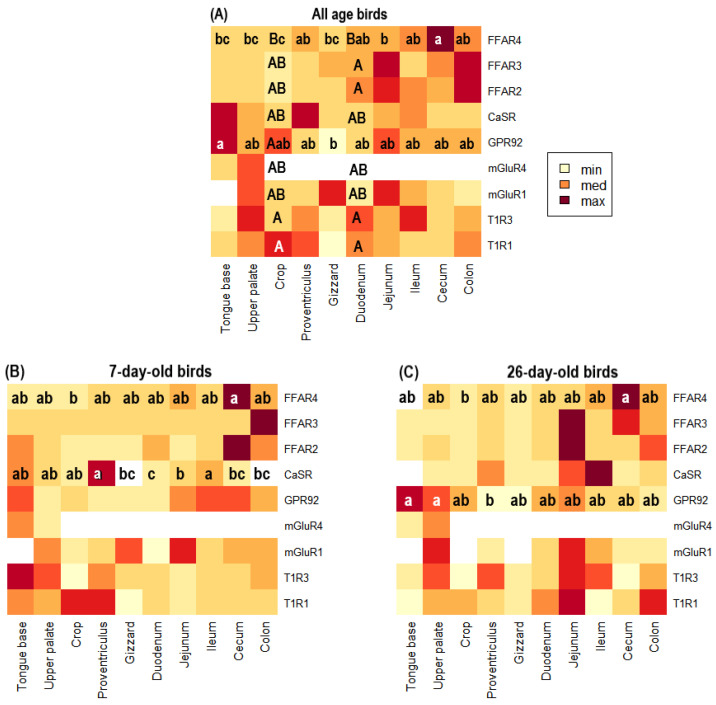
Heatmaps representing the level of expression of T1R1, T1R3, mGluR1, mGluR4, GPR92, CaSR, FFAR2, FFAR3, and FFAR4 in ten sections of the GIT (tongue base, upper palate, crop, proventriculus, gizzard, duodenum, jejunum, ileum, cecum, and colon) of broiler chickens. (**A**) Expression of genes of interest by GIT tissue averaging 7 and 26-day-old broilers. (**B**) Expression of genes of interest by GIT tissue in 7-day-old broilers. (**C**) Expression of the genes of interest in 26-day-old broilers. Color intensity indicates the level of expression, whitish being the lowest expression (min = low expression) and maroon the highest expression (max = high expression). Different letters (a, b, and c) mean significant differences in gene expression within a row (*p* ≤ 0.050). Different letters (A, B) mean significant differences in gene expression within a column (*p* ≤ 0.050).

When considering 7 and 26-day-old chickens no differences were observed in the expression of sensors at the levels of the tongue base, upper palate, proventriculus, gizzard, jejunum, ileum, cecum, and colon (*p* > 0.050). In the crop, a higher expression of T1R1, T1R3, and GPR92 than FFAR4 was registered (*p* = 0.005) and in duodenum a higher expression of T1R1, T1R3, FFAR2, and FFAR3 than FFAR4 was recorded (*p* = 0.003). The analysis of each gene between tissues showed a higher expression of FFAR4 in cecum compared to jejunum and crop (*p* < 0.01). It was also shown that GPR92 was expressed more in tongue base than in gizzard (*p* < 0.01; Figure 3A). When considering 7-day-old chickens, a higher expression of CaSR was observed in the proventriculus and ileum than in the jejunum and duodenum (*p* = 0.018) and FFAR4 was higher in the cecum than in the crop (*p* = 0.024; Figure 3B). Finally, when considering 26-day-old chickens, a higher expression of GPR92 was observed in the tongue base and upper palate than in the gizzard (*p* = 0.005) and FFAR4 was higher in the cecum than in the crop (*p* = 0.004; Figure 3C).

### 3.4. Correlations between Gene Expressions

**Figure 4:**
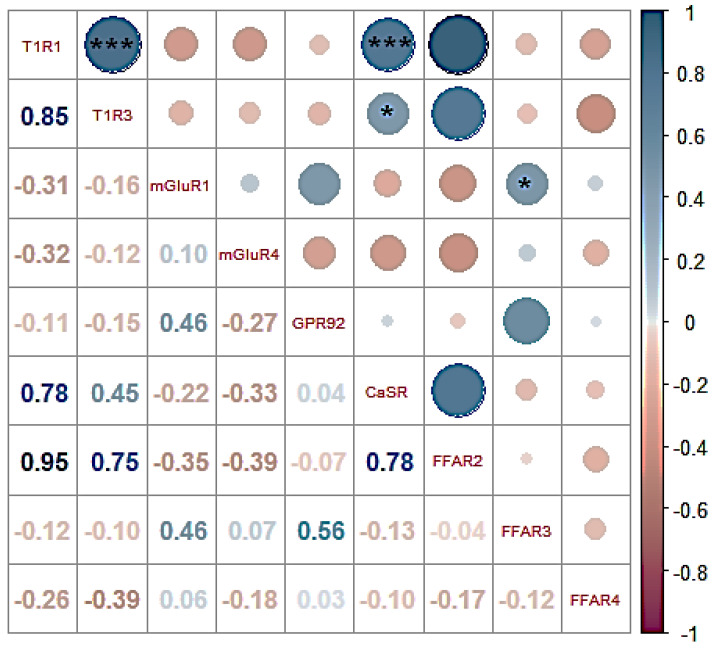
Correlogram of T1R1, T1R3, mGluR1, mGluR4, GPR92, CaSR, FFAR2, FFAR3 and FFAR4 sensors expression in broiler chickens. Values of the Spearman correlation coefficient for each comparison are shown on the left. (*, ***) = significant values (*p* < 0.050 and *p* < 0.001, respectively).

For the correlogram, the expression values were calculated using the average expression of each gene in all the tissues evaluated (Figure 4). Positive correlations were registered between the expression of T1R1 and T1R3 (r = 0.85, *p* < 0.001), CaSR and T1R1 (r = 0.78, *p* < 0.001), CaSR and T1R3 (r = 0.45, *p* < 0.050), and mGluR1 and FFAR3 (r = 0.46, *p* < 0.050)

## 4. Discussion

**Figure 5:**
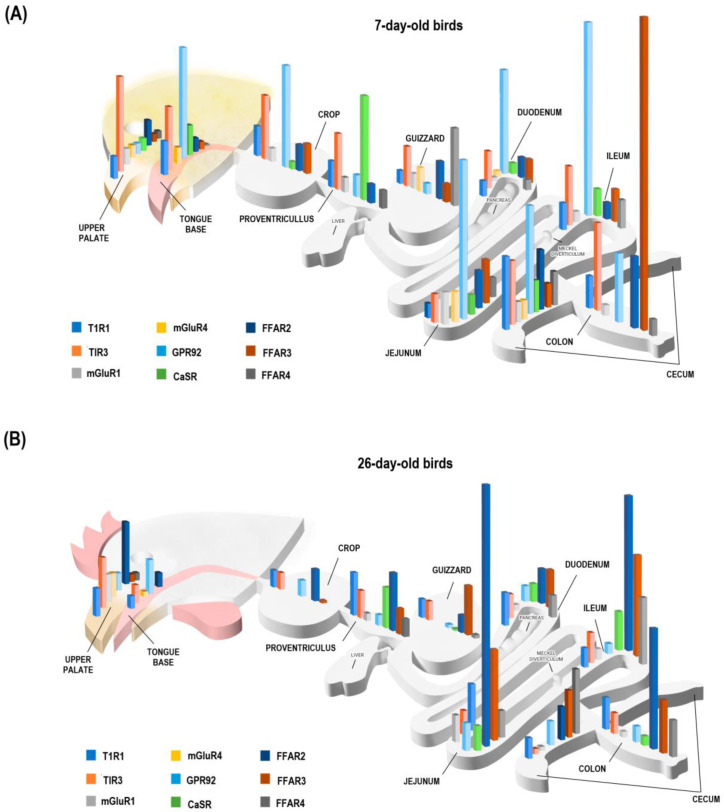
Gene expression of T1R1, T1R3, mGluR1, mGluR4, GPR92, CaSR, FFAR2, FFAR3, and FFAR4 sensors in ten sections of the GIT (tongue base, upper palate, crop, proventriculus, gizzard, duodenum, jejunum, ileum, cecum, and colon) of broiler chickens, (**A**) in 7-day-old birds (**B**) and 26-day-old birds.

The GIT monitors intestinal contents through transmembrane receptors (including nutrient sensors) located in the apical membrane of epithelial cells [19]. Some of these cells, particularly of the enteroendocrine system, regulate hormonal secretion and intestinal motility, mediating consumption, digestion, and absorption of nutrients [8,20]. In birds, little is known about sensors that respond to dietary AA and FA [20,21]. This study provides an exhaustive and comprehensive analysis of the expression of AA and FA sensors throughout the GIT of broiler chickens at two different ages (7 and 26 days post-hatching). A global scheme of their expression is presented in Figure 5.

Changes in the expression of nutrient sensors at different ages of birds are closely associated with the functional role of these receptors, which in turn determines a higher density of certain genes in specific sections of the GIT. These findings at different ages indicated a relationship with the physiological demands of birds during their development, showing at seven days of life the primary density of AA sensors in the upper GIT, particularly the oral cavity, suggesting a greater relevance for promoting feed intake through taste sensing when the growth relative to body weight is maximal (e.g., 7-day-old chicks) followed by a lower expression of AA in 26-day-old chickens compatible with a slower growth relative to body weight than the first days of life. Moreover, in the early stages of life, there is greater expression of FA in the lower GIT, particularly the cecum (FFAR2 and 4) and colon (FFAR3), which could be related to the establishment of a healthy microbiota and his association with sensing in the lower gut. At 26 days of the life of the birds, a significant presence of sensors is maintained (but decreased) in the oral cavity, cecum, and colon. An important change to highlight is the increase in AA and FA sensors in the jejunum, which is compatible with its main role in controlling food intake and nutrient absorption to maximize development, specifying its maximum growth at that age. It should be noted that previous studies in birds have already reported the expression of nutrient sensors and taste signaling molecules in the GIT with variations according to age [8,22,23], and an example of this is what was found by Dong (2020) who reported high T1R1 gene abundance in birds after hatching that decreased in 21-day-old chickens. Moreover, it has been reported that the type of diet influences the expression of nutrient sensors, and it has been determined that a high-protein diet induces high levels of expression of AA sensors [24]. This is consistent with the dietary profile supplied during the first days of the life of chickens, which includes high protein and AA levels to satisfy the nutritional requirements for optimal growth. Regarding the effect of diet on the expression of sensors, it could be determined by a local paracrine cellular regulation that responds to the presence and contact of nutrients with luminal intestinal cells [25]. The high expression of FA sensors in the lower GIT of 7 and 26-day-old birds could be explained given the considerable number of microorganisms conforming to the intestinal microbiota with essential functions in diet breakdown being linked to nutrient-sensing cells [26,27]. The premise gains support from the fact that the microbiota exerts a direct influence on nutrient detection mechanisms, specifically on the gut-brain axis, indicating that it would upregulate GPCRs [28], which is consistent with a greater FA sensors abundance associated with the local bacterial fermentation machinery [29]. The expression of AA sensors in the upper tract of birds has been related to short-term intake control mechanisms since it has been described that oral AA have an orexigenic effect that induces higher food intake by stimulating the taste cortex [1,19,30]. This high expression of sensors is also associated with releasing digestive agents and hormones, such as cholecystokinin, at the stomach level [1,30].

Tissue-specific genetic analysis showed a greater abundance of GPR92 in the oral cavity (tongue base and upper palate) in 26-day-old chickens, which is consistent with the literature reporting its presence in large populations of taste buds in type II cells of the murine model [31] and in chicken GIT cells [7]. This receptor is also known as LPA5 (G12/13 and Gq-coupled lysophosphatidic acid receptor), given its essential function as an intracellular signaling agent in the foregut as a sensor of partially digested proteins [24,32], which justifies its presence in extra-gustatory tissues as observed in this research. Previous findings have associated the presence of CaSR with an important AA and calcium-sensing mechanism [7]. In this study, the high CaSR expression in the proventriculus of 7-day-old birds could be attributed to the high demands for tissue conformation and growth rate in fattening. It is important to note that this study is the first to analyze CaSR throughout the broilers’ GIT, considering that previous reports were conducted in layers [13,33]. The high expression of FFAR2 and FFAR3 in tissues of the lower GIT section responds to their association with sensing of short-chain fatty acids (SCFA) produced by bacterial fermentation [34] and specifically in birds, the cecum are the main fermentation organs associated with the densest and most diverse microbiota [35,36]. Likewise, the greater expression of these genes at the level of the middle tract of birds is related to feedback mechanisms SCFA that mediate the rate of passage and hormone release, such as GLP-1 and PYY [37,38]. The expression of FFAR4 in tissues of the lower GIT of birds is not only associated with the detection of long-chain FA, but it also fulfills a regulatory function of the immune response, leading to the production of cytokines by the cells of the intestinal epithelium [39,40].

In this work, we observed an expected positive correlation between T1R1 and T1R3 since both genes join to form the main heterodimer that senses AA from diets [41]. Positive correlations were also observed between CaSR and T1R1/T1R3, which could be explained by the high synchrony required for amino acids detection during a feeding event, suggesting synergism in their function. On the other hand, the expressions of GPR139 and GPRC6A were not detected in this study, probably due to very low expression in the GIT tissues of chickens. Since their expression has been previously reported in rodents [7,20], it would be justifiable to conduct future research to corroborate and elucidate their presence and function in birds. Finally, it is important to consider that the transcriptomic results from this study should be interpreted with caution due to the constraint that not all transcribed mRNA will be translated into functional proteins.

## 5. Conclusions

Early in life (7 days), chickens show a high level of expression of AA sensors in the oral cavity (upper GIT section), co-expressed with high levels of FFAR2, 3, and 4 mainly in the cecum (FFAR2 and 4) and colon (FFAR3). Thus, at early stages in the life of a chicken, the nutrient-sensing profiles seem to promote oral sensing (thus potentially feed intake) and the establishment of sensing capacity around the main site of microbial fermentation, the lower gut. Later in the life of the chicken (26 days), a significant presence of nutrient sensors emerges in the jejunum, making the GIT section the main sensing site for nutrients, including both AA and FFAR sensors. This is compatible with the principal role of jejunum in the control of feed intake and nutrient absorption.

## Figures and Tables

**Table 1:** Composition and chemical analysis of the starter and grower diets used in the experiment.

Item	Starter	Grower
Ingredients (g/kg)		
Corn	554.2	630.9
Soybean meal (47% protein)	270.3	192.5
Ground wheat	50.0	50.0
Rapeseed meal	40.0	70.0
Gluten meal (60% protein)	30.0	0
Olein oil	16.5	20.3
CaCO_3_	14.4	16.3
CaHPO_4_	11.8	7.1
NaCl	4.4	4.1
Lys	2.2	1.6
Met	2.1	3.2
Micofix plus ^1^	0.05	0.05
Coccidiostat	0.5	0.5
Multivitamins–mineral–phytase ²	2.0	2.0
Formicit dry ^3^	1.0	1.0
Analyzed nutrient composition (%)		
Dry matter	88.9	88.7
Crude protein	22.9	18.1
Crude fiber	3.4	4.3
Ether extract	3.8	4.9
NNE	53.7	55.0
Ash	5.1	6.4

¹ Integral solution for mycotoxins (Virbac Centrovet, Santiago, Chile); ² Contains per kilo of premix: 8000 UI of Vit. A, 2500 UI of Vit. D3, 15 UI of Vit. E, 1.5 mg of K3, 1.5 mg of Vit. B1, 5 mg of Vit. B2, 35 mg of niacin, 13.1 mg of calcium pantothenate, 2.49 mg of Vit. B6, 0.012 mg of Vit. B12, 1 mg of folic acid, 0.1 mg of biotin, 399.5 mg of choline, 25 mg of Fe, 70 mg of Mn, 60 mg of Zn, 6 mg of Cu, 0.15 mg of Se, 100 mg of antox, 0.5 mg of I, 100 mg of Hostazym X, 50 mg of Optiphos G; ³ Preservative anti-salmonella sp. for food preservation (Veterquímica SA, Santiago, Chile).

**Table 2:** Primers used in the study for mRNA analysis ^1^.

Gene	GenBank Accession No ^2^	Forward Primer (5′ → 3′)	Reverse Primer (3′ → 5′)
T1R1	XM_015297117.1	CTA TGG TAG GGA TGG GCT CAA C	CTA AAG ACC AGT CCT CAG AGC C
T1R3	KM091452	GTC TTC GCC ACT CTG AGG AC	CTA AAG ACC AGT CCT CAG AGC C
mGluR1	NC_052534.1	CGC GCC AGG TTA AAA GTC AC	GGT TCC TCT TGG CTG CGT AT
mGluR4	NC_052557.1	GTG CAA GCC CTG ATT GAG AAG	GTG GAG GCA TAG CTG ATC TGG
CaSR	XM_416491.5	TGG CTT CCA CCT TGT TGC TTA	GCA GCA GTG TTC CAG GTA AAC
GPR139	NM_001321735.1	TGC TGA CAT CCT CGT TCT CTT	GAG TGG ATG GCA CAC AGC TA
GPRC6A	XM_426177	GGA GGT TTG TTT GCA GTT CAC A	TTT GGA CAG GAA CCT CAG AGC
GPR92	XM_015293753.1	GGA CAA ACC TGG CAC TCA GA	GCT AGG GGC TTT CTG TGG TT
FFAR2	GCF_000002315.4	GCC CCA TAG CAA ACT TCT	GGG CAG CCA TAA AGA GAG
FFAR3	JQ927550.1	GAA GGT GGT TTG GGA GTG AA	CAG AGG ATT TGA GGC TGG AG
FFAR4	XM_040675455.1	GCA CGG ACA GAA GGA AGA AG	CCA CCC CTG AAG TCT GAG AA
β-actin	NM_205518.1	GAG AAA TTG TGC GTG ACA TCA	CCT GAA CCT CTC ATT GCC A

¹ Primers were designed using Primer3Plus (version 2.3.1, Free Software Foundation, Boston, MA, USA); ^2^ Reference chicken gene sequence that contains the corresponding PCR products list.

## Data Availability

The data are available upon reasonable request to the submitting author.
